# Andrographolide Attenuates Sepsis-Induced Acute Lung Injury *via* Peroxisome Proliferator-Activated Receptor Gamma in a Murine Cecal Ligation and Puncture Model

**DOI:** 10.2174/0118715303418976251204115210

**Published:** 2026-03-27

**Authors:** Lokesh V. Thimmana, Hari Prakash Kalla, Kumarla Kaluva Ruhinaz, Gangadri Byalla, Kalpana Panati, Venkata Ramireddy Narala

**Affiliations:** 1 Department of Zoology, Yogi Vemana University, Kadapa, India;; 2 Department of Microbiology, Yogi Vemana University, Kadapa, India;; 3 Department of Biotechnology, Government College for Men, Kadapa, India

**Keywords:** Acute lung injury, andrographolide, PPARγ, inflammation, microbial sepsis, acute respiratory distress

## Abstract

**Introduction:**

Sepsis-induced acute lung injury (ALI) is a life-threatening complication marked by intense inflammation, oxidative stress, and respiratory failure, with no effective targeted treatments available. This study aims to explore the protective effects of andrographolide (AGL) against sepsis-induced ALI and uncover its underlying mechanisms, focusing on the modulation of peroxisome proliferator-activated receptor gamma (PPARγ) as a potential therapeutic pathway.

**Methods:**

A murine model of ALI was established using cecal ligation and puncture (CLP). AGL was administered to evaluate its effects on pulmonary inflammation and oxidative stress. Neutrophil and macrophage infiltration, pulmonary edema, and levels of malondialdehyde and nitrite were measured in bronchoalveolar lavage (BAL) fluid and lung tissue, along with the expression of PPARγ and the activities of antioxidant enzymes superoxide dismutase (SOD) and catalase. The involvement of PPARγ was further confirmed using GW9662, a selective PPARγ antagonist.

**Results:**

AGL treatment significantly attenuated neutrophil and macrophage infiltration, reduced pulmonary edema and inflammation, and decreased nitrite levels in BAL. Additionally, AGL upregulated PPARγ expression and enhanced SOD and catalase activity. These protective effects were significantly reversed by co-administration of GW9662, indicating a PPARγ-dependent mechanism.

**Discussion:**

The present study demonstrated that AGL exerts significant protective effects against CLP-induced ALI, primarily through modulation of inflammatory and oxidative pathways.

**Conclusion:**

AGL’s ability to upregulate PPARγ and enhance SOD and catalase activity further supports its mechanistic role in mitigating lung damage. The reversal of AGL protective effects by GW9662 confirms its PPARγ-dependent mechanism, highlighting its potential as a targeted protective treatment for sepsis-induced ALI.

## 1 INTRODUCTION

Sepsis-induced acute lung injury (ALI) remains a critical clinical challenge, characterized by excessive inflammation, oxidative stress, and disruption of pulmonary architecture, often culminating in acute respiratory distress syndrome and high mortality [1, 2]. Among the various experimental models, cecal ligation and puncture (CLP) is a widely accepted method to mimic the complex pathophysiology of polymicrobial sepsis and ALI [3, 4]. The pathogenesis of CLP-induced ALI involves extensive infiltration of inflammatory cells, protein leakage into the alveolar space, and elevated expression of pro-inflammatory cytokines such as interleukin-1β (IL-1β) and interleukin-6 [5, 6]. Sepsis-induced ALI currently lacks targeted therapies, with management relying predominantly on supportive care. Given its high mortality rate, there is a pressing need for novel treatment strategies [7, 8].

Andrographolide (AGL), a bioactive diterpenoid lactone derived from *Andrographis paniculata*, has garnered attention for its anti-inflammatory and antioxidant properties [9, 10]. Previous studies have demonstrated that AGL can inhibit NLRP3 inflammasome activation and attenuate pulmonary inflammation in models of chronic obstructive pulmonary disease [5]. Recent studies have indicated that AGL alleviates sepsis-induced ALI by promoting autophagy in alveolar macrophages *via* inhibition of the RAGE/PI3K/AKT/mTOR pathway, thereby suppressing NLRP3 inflammasome activation and reducing inflammation [11, 12]. Additionally, AGL and dehydroandrographolide [13], a derivative of AGL, have shown efficacy in reducing lung edema and inflammation in preclinical ALI models [14-16]. This highlights AGL’s potential as a therapeutic agent targeting immune homeostasis in acute lung injury. However, the role of AGL in modulating PPARγ signaling pathways during sepsis-induced ALI remains to be elucidated.

Recent evidence suggests that its pharmacological actions may be mediated through modulation of nuclear receptors, particularly PPARγ, a key regulator of inflammation and immune responses [17, 18]. PPARγ, a nuclear receptor and transcription factor, plays a pivotal role in modulating inflammatory responses and maintaining immune homeostasis [19, 20]. Activation of PPARγ has been shown to suppress nuclear factor-kappa B (NF-κB) signaling, thereby reducing the expression of pro-inflammatory mediators [21, 22]. Moreover, PPARγ activation upregulates antioxidant enzymes, such as heme oxygenase 1 (HO1) and Nrf2, and reduces oxidative stress in lung tissues [23-25]. These properties underscore the therapeutic potential of PPARγ agonists in treating inflammatory lung diseases. However, the mechanistic role of AGL in CLP-induced lung inflammation, especially through the PPARγ signaling axis, remains to be fully elucidated.

In this study, we investigated the protective effects of AGL in a murine model of CLP-induced ALI. Specifically, we assessed its ability to attenuate lung inflammation, suppress inflammatory cell infiltration and protein leakage into the BAL fluid, and regulate the expression of oxidative stress and inflammatory markers. Moreover, we examined whether these protective effects were mediated by PPARγ using the specific PPARγ antagonist GW9662. Our findings provide compelling evidence that AGL exerts its anti-inflammatory effects in CLP-induced lung injury through a PPARγ-dependent mechanism.

## 2 EXPERIMENTAL METHODS

### 2.1 Cecal Ligation and Puncture (CLP)-Induced Acute Lung Injury in Mice

Healthy female C57BL/6 mice (6–8 weeks old, approximately 20 grams) were procured from Mahaveera Enterprises, Hyderabad, India, and used to induce acute lung injury *via* CLP. Mice were randomly divided into five groups (n = 5 per group), with each group housed separately and food and water provided *ad* libitum. Efforts were made to minimize animal suffering. The CLP procedure was performed to include ALI, followed by subcutaneous resuscitation with normal saline (0.9% NaCl) as previously described [26, 27]. Briefly, under anesthesia (ketamine 100 mg/kg and xylazine 10 mg/kg, i.p.), the cecum was ligated and punctured through-and-through with a 22-*g* needle to allow polymicrobial leakage, mimicking human sepsis-induced lung injury.

#### 2.1.1 Experimental Design

The study included five groups of animals, organized as follows:

Group 1 (Control + Vehicle): Sham-operated animals received Phosphate-Buffered Saline (PBS) as a vehicle control.

Group 2 (CLP + Vehicle): Animals underwent cecal ligation and puncture (CLP) and received PBS.

Group 3 (CLP + AGL): Animals were pretreated with AGL for 2 days at a dose of 20 mg/kg body weight, administered intraperitoneally (i.p.) in a volume of 200 µl. AGL (98% purity; Catalogue #365645, Lot #MKCR6062; Sigma Aldrich, India) was administered twice daily for two consecutive days and one dose prior to CLP induction. The dose and route of administration were selected based on previous studies [28-31].

To investigate whether the effects of AGL are mediated *via* PPARγ, a separate experiment was conducted with two additional groups:

Group 4 (CLP + GW9662): Animals received GW9662 (1 mg/kg, i.p.), a selective PPARγ antagonist, twice daily for two consecutive days and one dose prior to CLP induction.

Group 5 (CLP + GW9662 + AGL): Animals were pretreated with GW9662 (1 mg/kg, i.p.) 30 minutes before receiving AGL (20 mg/kg, i.p.) as described above.

This design allowed for the evaluation of AGL’s protective effects and the role of PPARγ in mediating those effects. Mice were euthanized by CO_2_ asphyxiation 4 hours after CLP to assess various parameters associated with ALI. The 4-hour time point was chosen to capture the early inflammatory phase of polymicrobial sepsis, a widely accepted model for this purpose [26, 27]. All animal procedures were approved by the Institutional Animal Ethics Committee at Yogi Vemana University (YVU-IAEC-NVRR-01-2023) and were conducted in accordance with the Committee for the Control and Supervision of Experiments on Animals (CCSEA), New Delhi, India, as well as the NC3Rs ARRIVE guidelines [32].

### 2.2 Bronchoalveolar Lavage (BAL) Fluid Collection and Analysis

BAL fluid was collected by flushing the lungs three times with 1 ml of PBS *via* a tracheal catheter. The fluid was centrifuged at 400 × g for 10 minutes at 4°C, and the supernatant was stored at −80°C for further analysis. The cell pellet was resuspended in 1 ml of PBS, and total cell counts were determined using a hemocytometer, followed by Giemsa staining (Sigma-Aldrich, India) for differential cell counts, as described previously [33, 34]. Total protein concentration in the BAL fluid was measured using the Lowry method [35].

### 2.3 Histological Examination of the Lungs

Following euthanasia, lung tissues were fixed in 10% neutral buffered formalin, embedded in paraffin, and sectioned at 5 μm thickness. Sections were stained with Hematoxylin and Eosin (H&E) for histological analysis [36]. Lung injury was evaluated in a blinded manner based on peribronchiolar infiltrates, hemorrhage, alveolar cellular infiltration, and edema, as described in previous studies [37, 38].

### 2.4 Lung Wet/Dry Weight Ratio

To assess pulmonary edema, lungs were collected post-euthanasia, blotted, and weighed for wet mass. Samples were then oven-dried at 60°C for 48 hours to obtain dry weight. The wet-to-dry (w/d) weight ratio was calculated to quantify fluid accumulation in lung tissue as described previously [39].

### 2.5 Oxidative Stress Marker Analysis

Superoxide Dismutase (SOD) activity was measured using a previously described method [40], with absorbance read at 543 nm. Results were expressed as units of SOD activity per milligram of protein. Catalase activity was assessed according to a standard protocol [41] and expressed in katal per second per milligram of protein. Lipid peroxidation in lung tissue was evaluated by quantifying Malondialdehyde (MDA) levels using a modified version of a previously established method [42]. MDA concentration was determined by measuring the absorbance of the upper phase at 532 nm and reported as nanomoles per milligram of protein. Serum nitrite levels, used as an indirect marker of nitric oxide production, were measured following a previously described protocol [43]. Concentrations were determined using a sodium nitrite standard curve and expressed as μM/ml of serum.

### 2.6 RNA Extraction and RT-PCR

Total RNA was extracted from lung tissue using the RNeasy^®^ Mini Kit (Qiagen) according to the manufacturer’s instructions. RNA concentration and purity were assessed using a NanoDrop 2000 spectrophotometer (Thermo Fisher Scientific). One microgram of total RNA was reverse-transcribed into cDNA using the iScript™ cDNA Synthesis Kit (Bio-Rad, India) [44]. Semi-quantitative PCR was performed using a thermal cycler (Applied Biosystems) with Hi-Chrome PCR Master Mix (Himedia, India). PCR products were resolved on a 1.5% agarose gel stained with ethidium bromide. Gene expression levels were normalized to 18s rRNA, and the specific primers used are listed further.

PPARγ- forward- 5’-TCTCTCCGTAATGGAAGACC-3’, reverse- 5’-GCATTATGAGACATCCCCAC-3’,

HO1- forward- 5’- TGAAGGAGGCCACCAAGGAGG-3’, reverse- 5’- AGAGGTCACCCAGGTAGCGGG-3’,

Nrf2 - forward- 5’- AAGAATAAAGTCGCCGCCCA-3’, reverse- 5’- AGATACAAGGTGCTGAGCCG-3’

IL1β forward- 5’- GAAATGCCACCTTTTGACAGTG-3’, reverse- 5’- TGGATGCTCTCATCAGGACAG-3’,

18s rRNA, forward- 5’-ACCTGGTTGATCCTGCCAGTAG-3’

reverse-5’-TTAATGAGCCATTCGCAGTTTC-3

### 2.7 Statistical Analysis

Statistical significance was determined using one-way ANOVA followed by Tukey’s post hoc test for multiple comparisons. Analyses were performed using GraphPad Prism (San Diego, CA), with *p* < 0.05 considered statistically significant.

## 3 RESULTS

### 3.1 AGL Treatment Attenuates CLP-Induced Inflammatory Cell Infiltration, Protein Leakage, Pulmonary Edema, and Histological Lung Injury

**Fig. (1):**
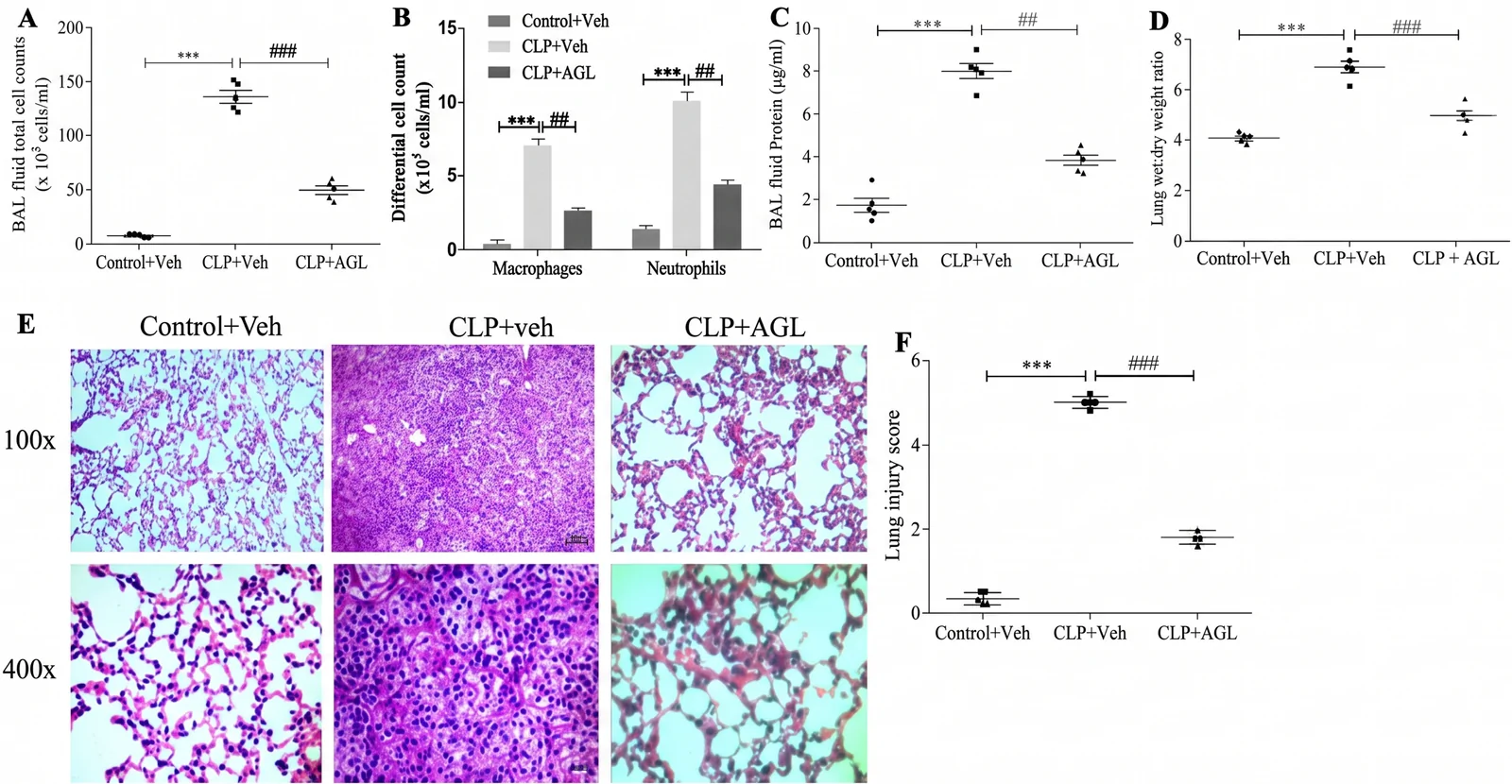
Andrographolide (AGL) reduces CLP-induced inflammatory cell infiltration, lung inflammation, alveolar-capillary leakage, and lung inflammation. (**A**) Total cell counts in BAL fluid from control (vehicle (veh)-treated), CLP, and CLP+AGL-pretreated (20 mg/kg) mice. (**B**) Differential cell counts showing macrophages and neutrophils in BAL fluid. (**C**) Total protein concentration in BAL fluid, indicative of alveolar-capillary barrier disruption. (**D**) Lung wet:dry weight ratio. (**E**) Representative hematoxylin and eosin (H&E) stained lung sections showing histopathological changes across groups. Magnifications: ×100 (top panel), scale bar = 10 μm, and ×400 (bottom panel); scale bar = 2.5 μm. (**F**) Quantitative assessment of lung injury scores based on histological criteria. Data are presented as mean ± SE (n = 5 mice per group). Statistical significance was determined by one-way ANOVA followed by Tukey's post hoc test. ***p < 0.001 *vs*. Control; ^##^*p* < 0.01 *vs*. CLP group; ^###^*p* < 0.001 *vs*. CLP group.

To assess the anti-inflammatory and protective effects of AGL, BAL fluid and lung tissue were analyzed following CLP. As shown in Fig. (**1A**), total BAL fluid cell counts were significantly increased in the CLP group compared to the control group (*p* < 0.001), indicating marked inflammatory cell infiltration. Treatment with AGL significantly reduced total cell counts compared to the CLP group (*p* < 0.001). Differential cell analysis revealed that both macrophage and neutrophil counts were markedly elevated in the CLP group relative to control (*p* < 0.001), whereas AGL treatment significantly decreased their levels (*p* < 0.001 for both cell types; Fig. **1B**). BAL fluid protein concentrations, a marker of vascular permeability and lung injury, were significantly increased in CLP mice compared to controls (*p* < 0.001; Fig. **1C**). AGL treatment significantly attenuated this protein leakage (*p* < 0.01), indicating reduced alveolar-capillary barrier disruption. Furthermore, analysis of the lung w/d ratio mirrored the trends observed in BAL fluid, showing a significant reduction in the w/d ratio with AGL treatment in CLP mice, an indication of reduced pulmonary edema (Fig. **1D**).

Histopathological analysis of lung tissues revealed severe alveolar wall thickening, inflammatory cell infiltration, and hemorrhage in CLP mice (Fig. **1E**). In contrast, lungs from AGL-treated mice displayed relatively preserved alveolar architecture with minimal cellular infiltration. Quantitative lung injury scoring (Fig. **1F**) confirmed a significant increase in lung injury in CLP animals compared to controls (*p* < 0.001), which was significantly ameliorated by AGL treatment (*p* < 0.001).

### 3.2 AGL Regulates Oxidative Stress and Inflammatory Gene Expression in CLP-Induced Lung Injury

**Fig. (2):**
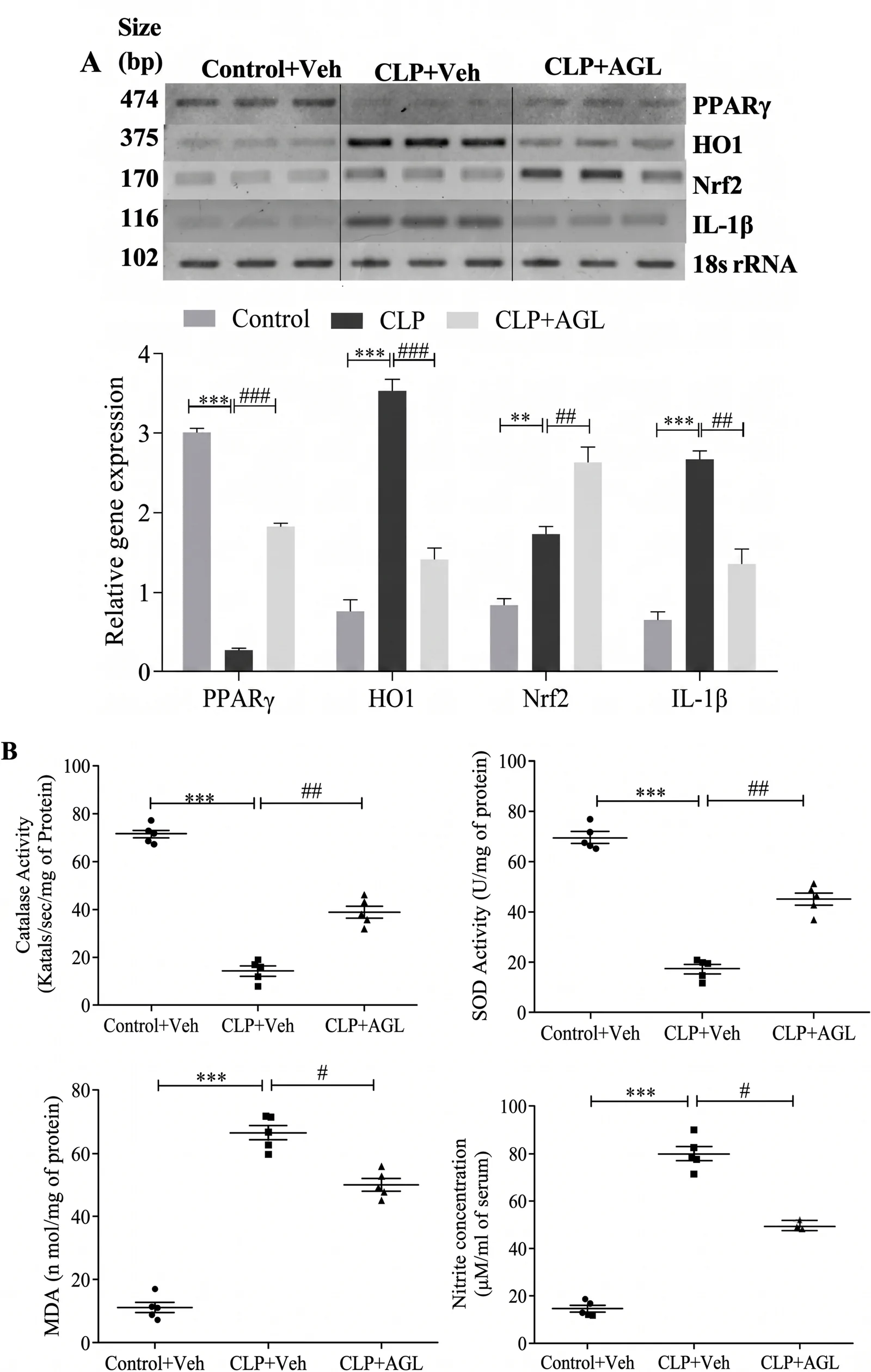
Andrographolide (AGL) modulates oxidative stress and inflammatory gene expression in acute lung injury induced by CLP. (**A**) Representative gel electrophoresis images showing mRNA expression levels of PPARγ, HO1, Nrf2, IL-1β, and 18s rRNA across three experimental groups and corresponding densitometric analysis (bottom) of mRNA expression levels normalized to 18s rRNA. (**B**) Antioxidant enzyme (catalase and superoxide dismutase (SOD) activity), and oxidative stress markers (Malondialdehyde (MDA) and serum nitrite levels), bottom panel. Data are presented as mean ± SE (n = 5 per group). Statistical comparisons were made using one-way ANOVA followed by Tukey’s post hoc test. Veh, vehicle treated. **p < 0.01 *vs*. Control, ***p < 0.001 *vs*. Control; ^#^*p* < 0.05, ^##^*p* < 0.01, ^###^*p* < 0.001 *vs*. CLP.

To elucidate the mechanisms underlying the protective effects of AGL in CLP-induced lung injury, we assessed the expression of key oxidative stress-related and inflammatory genes, as well as associated enzymatic and biochemical markers. RT-PCR analysis revealed a marked downregulation of PPARγ mRNA levels in lung tissues from CLP mice compared to controls (Fig. **2A**). Conversely, the expression of the pro-inflammatory cytokine IL-1β and antioxidant target genes Nrf2 and HO1 was significantly upregulated in the CLP group (*p* < 0.01). AGL treatment significantly restored PPARγ expression (*p* < 0.001), further increased Nrf2 levels, and concurrently reduced the expression of HO1 and IL-1β compared to the CLP group (*p* < 0.01), indicating a transcriptional shift towards anti-inflammatory and antioxidant responses (Fig. **2A**).

We further evaluated antioxidant defense mechanisms and oxidative stress biomarkers. As shown in Fig. (**2B**), CLP caused a significant decrease in catalase and SOD activities relative to control (*p* < 0.001), reflecting compromised antioxidant capacity. AGL treatment significantly restored the activity of both enzymes (*p* < 0.01), suggesting improved oxidative defense. In addition, MDA levels, an index of lipid peroxidation, were markedly increased in CLP mice compared to controls (*p* < 0.001) and significantly reduced following AGL administration (*p* < 0.05). Similarly, serum nitrite levels, indicative of nitric oxide-mediated oxidative stress, were significantly elevated in the CLP group (*p* < 0.001) and attenuated by AGL treatment (*p* < 0.05) (Fig. **2B**). These findings demonstrate that AGL mitigates oxidative stress and inflammatory responses in CLP-induced lung injury, likely by modulating PPARγ and HO1 expression and restoring antioxidant enzyme activities.

### 3.3 PPARγ Antagonist GW9662 Reverses the Protective Effects of AGL in CLP-Induced Lung Injury

To determine the involvement of PPARγ in mediating the protective effects of AGL, mice were treated with the PPARγ antagonist GW9662 as described in the experimental design.

**Fig. (3):**
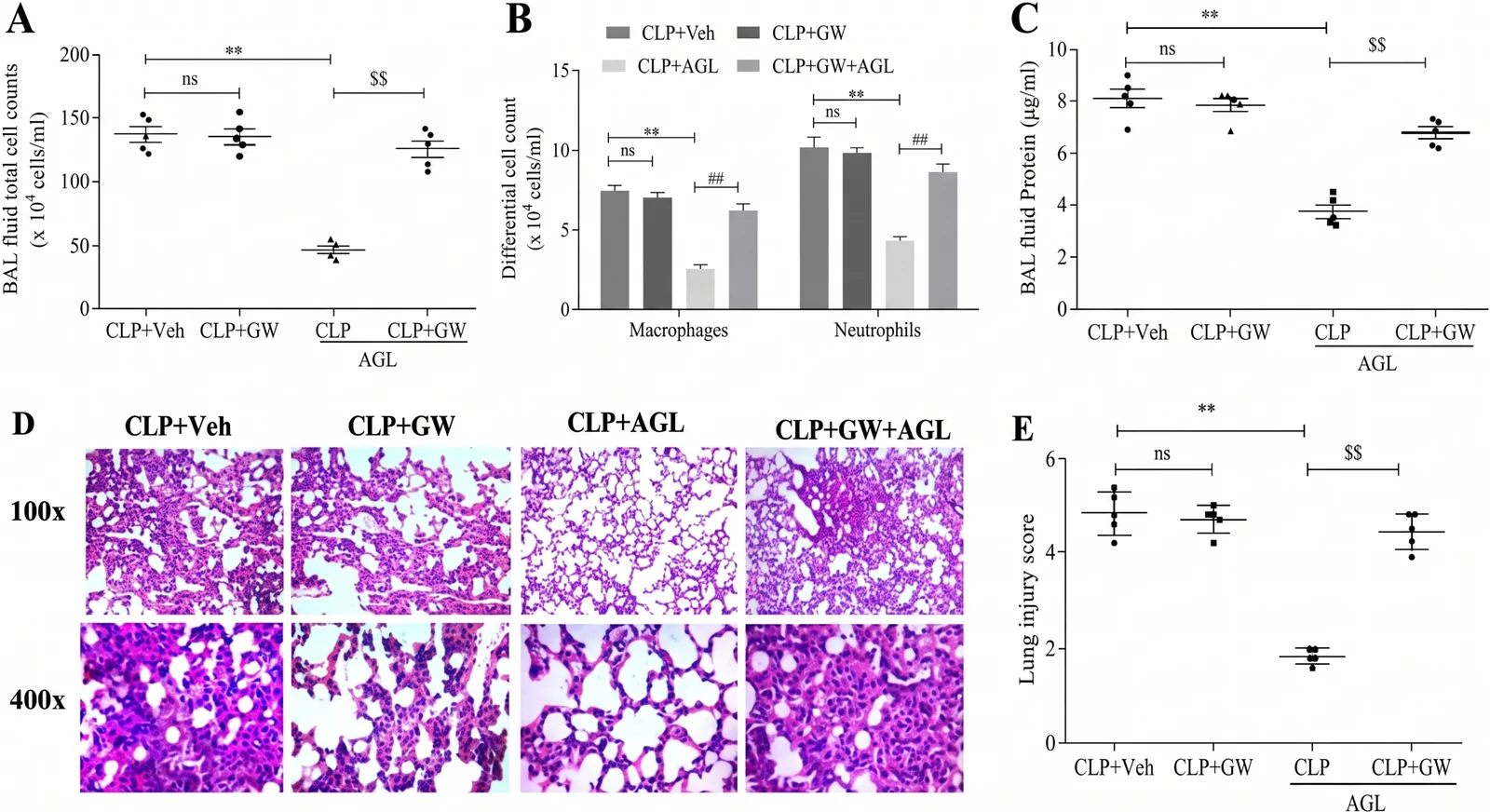
PPARγ antagonist GW9662 reverses the anti-inflammatory effects of AGL in CLP-induced lung injury. (**A**) Total cell counts in BAL fluid from CLP, CLP+GW9662 (1 mg/kg), CLP+AGL (20 mg/kg), and CLP+AGL+GW9662 (1 mg/kg) groups. (**B**) Differential cell counts in BAL fluid. (**C**) BAL fluid total protein content, indicating alveolar-capillary barrier disruption. (**D**) Representative H&E-stained lung tissue sections at 100× and 400× magnifications (scale bar = 10 µm and 2.5 µm, respectively), demonstrating lung histopathology. (**E**) Quantification of lung injury score based on the histological evaluation. Data are expressed as mean ± SE (n = 5 per group). Statistical significance was determined using one-way ANOVA followed by Tukey’s post hoc test. **p < 0.01 *vs*. CLP, ^$$^*p* < 0.01 *vs*. CLP+AGL, **Abbreviation:** ns. no significance.

As shown in Fig. (**3**), GW9662 had no observable effect on CLP-induced markers associated with the disease model. Treatment with AGL significantly reduced the total cell count in BAL fluid compared with the CLP group (*p* < 0.01). However, co-administration of GW9662 with AGL significantly reversed this reduction (*p* < 0.01), indicating a role for PPARγ in modulating inflammatory cell infiltration (Fig. **3A**). Differential cell count analysis (Fig. **3B**) revealed that both neutrophils and macrophages were markedly increased in the CLP group. AGL treatment significantly suppressed their infiltration (*p* < 0.01), whereas GW9662 treatment abrogated these effects (*p* < 0.01), restoring neutrophil and macrophage levels to levels comparable to those of the CLP group.

BAL fluid protein concentration, an indicator of alveolar-capillary barrier disruption, was also significantly reduced by AGL treatment relative to CLP (Fig. **3C**; *p* < 0.01). This protective effect was negated by GW9662, as protein levels significantly increased upon co-treatment (*p* < 0.01).

Histological examination of lung tissue (Fig. **3D**) demonstrated severe alveolar damage, inflammatory cell infiltration, and thickened alveolar septa in the CLP group. These pathological changes were markedly attenuated by AGL, whereas GW9662 co-treatment reversed the AGL-mediated improvements. Quantitative lung injury scores (Fig. **3E**) confirmed the histological findings. AGL treatment significantly reduced lung injury scores compared to CLP (*p* < 0.001), while co-administration of GW9662 significantly increased the score (*p* < 0.001), nearly restoring it to CLP levels. These findings suggest that the protective effects of AGL in sepsis-induced lung injury are at least partially mediated through activation of PPARγ signaling.

### 3.4 AGL Mitigates CLP-Induced Lung Injury *via* PPARγ Activation

**Fig. (4):**
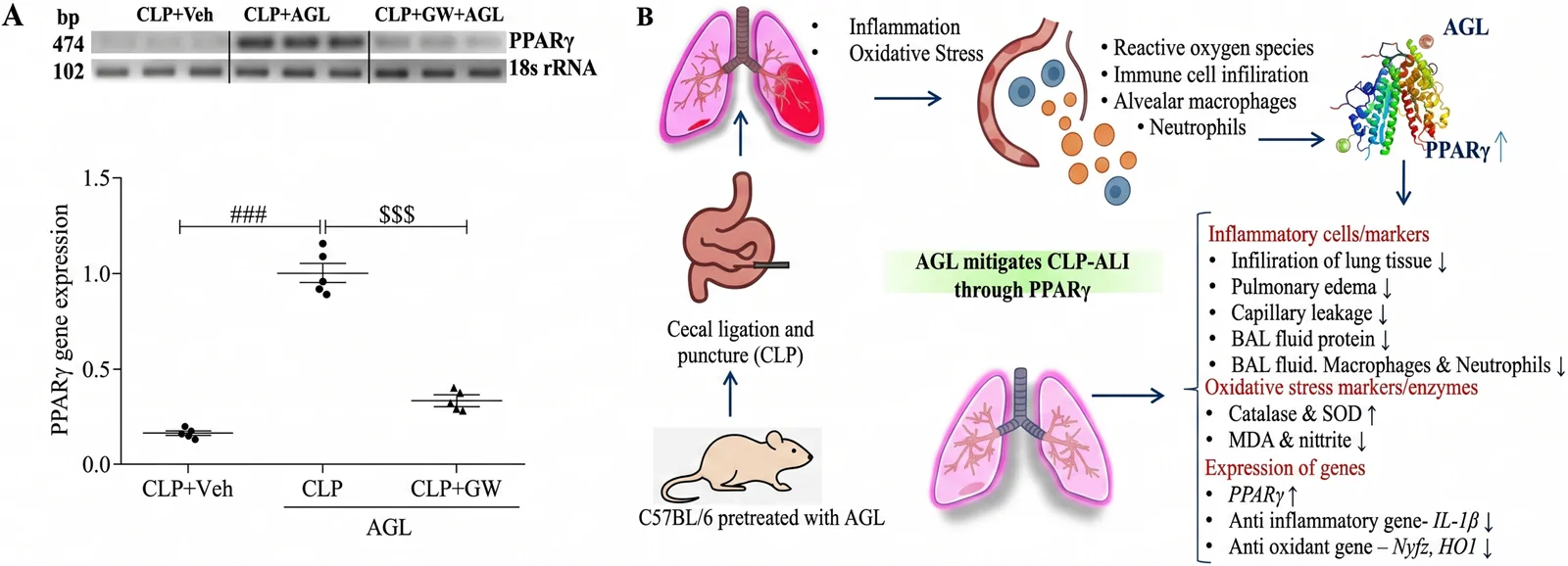
AGL mitigates CLP-induced lung injury *via* PPARγ activation. (**A**) Representative gel electrophoresis images showing mRNA expression of PPARγ in lung tissue from CLP, CLP+AGL (20 mg/kg), and CLP+AGL+GW9662 (1 mg/kg) groups and corresponding densitometric analysis normalized to 18s rRNA (bottom). Data are presented as mean ± SE (n = 5 per group); ^###^*p* < 0.001 *vs.* CLP, ^$$$^*p* < 0.001 *vs*. CLP+AGL. (**B**) Graphical summary showing AGL’s protective effects in CLP-induced acute lung injury (ALI) through PPARγ activation, including reduced inflammation, oxidative stress, and lung injury markers.

To confirm the involvement of PPARγ in the protective effects of AGL, we assessed PPARγ mRNA expression in lung tissue by RT-PCR. As shown in Fig. (**4A**), PPARγ expression was significantly upregulated in the CLP+AGL group compared to the CLP group (*p* < 0.01). However, co-administration of the PPARγ antagonist GW9662 significantly attenuated this AGL-induced upregulation (*p* < 0.01), indicating that AGL’s protective anti-inflammatory and antioxidant actions are mediated, at least in part, through PPARγ activation (Fig. **4B**).

## 4 DISCUSSION

In this study, we demonstrate that AGL exerts significant protective effects against sepsis-induced ALI in a murine model of CLP, which are mediated through PPARγ. AGL treatment markedly attenuated inflammatory cell infiltration, vascular protein leakage, and histopathological lung damage, indicating suppression of innate immune cell recruitment to the alveolar space. As neutrophilic inflammation drives tissue injury in sepsis-associated ALI, AGL may attenuate this by inhibiting chemokine production or leukocyte transmigration, consistent with effects seen in other inflammation models [45-47].

Additionally, reduced BAL fluid protein levels suggest that AGL preserves alveolar-capillary barrier integrity. Increased permeability and protein-rich edema fluid are central to the pathophysiology of ALI and correlate with disease severity and mortality [48, 49]. AGL’s ability to mitigate protein leakage suggests it may stabilize endothelial and epithelial tight junctions, thereby reducing pulmonary edema. Previous studies have reported that AGL exerts endothelial-protective effects by downregulating inflammatory signaling pathways such as NF-κB and MAPKs [14, 50], mechanisms that may underlie the present findings.

Histological assessment further substantiates AGL's anti-inflammatory and tissue-protective properties. Compared with the CLP group, AGL-treated lungs exhibited preserved alveolar architecture, reduced cellular infiltration, and lower injury scores. These results are consistent with earlier work showing that AGL alleviates LPS-induced ALI by modulating inflammatory and oxidative pathways [14]. Notably, recent evidence has implicated autophagy and inflammasome regulation in the therapeutic action of AGL in sepsis models, where it was shown to enhance autophagic flux in alveolar macrophages by inhibiting the RAGE/PI3K/AKT/mTOR axis and suppressing NLRP3 inflammasome activation [11]. Although not directly assessed in this study, it is plausible that similar mechanisms contribute to the protective effects observed.

PPARγ is a ligand-activated nuclear receptor with well-established anti-inflammatory and antioxidant properties [51, 52]. In our study, CLP significantly suppressed PPARγ mRNA expression in the lung, consistent with reports showing impaired PPARγ signaling during systemic inflammation and sepsis [53]. AGL administration restored PPARγ expression, suggesting that it may exert part of its protective effect *via* activation of PPARγ-dependent transcriptional programs. While the exact mechanism remains unclear, PPARγ ligands activate downstream genes and simultaneously prolong signaling by increasing PPARγ expression [54-56]. These findings align with earlier studies showing that AGL's protective effects are mediated through PPARγ in preclinical models [17, 18].

Similarly, HO1, a stress-inducible enzyme with cytoprotective and anti-inflammatory effects, was upregulated following CLP and was significantly downregulated with AGL treatment, as previously reported to exert anti-inflammatory and antioxidant effects [57]. Regulation of HO1 by other natural ligands of PPARγ has been previously reported in contexts of ovalbumin-induced airway inflammation [58], supporting its role in AGL-mediated cytoprotection in sepsis.

In addition, AGL significantly restored the activities of key antioxidant enzymes catalase and SOD, both of which were markedly reduced in CLP-induced lung injury. These enzymes play pivotal roles in scavenging ROS and preventing oxidative tissue damage [59, 60]. The restoration of catalase and SOD activities by AGL suggests that it preserves or reactivates endogenous antioxidant systems under septic conditions.

Furthermore, AGL markedly reduced the levels of MDA, a byproduct of lipid peroxidation, and serum nitrite levels, a surrogate marker of nitric oxide production and nitrosative stress, and attenuated these markers. AGL’s ability to lower MDA levels underscores its potential as a ROS scavenger or indirect modulator of oxidative stress through enzyme induction. The reduction in nitrite levels following AGL treatment suggests either direct suppression of iNOS expression or indirect inhibition *via* anti-inflammatory signaling, possibly mediated by PPARγ [61].

The present study demonstrated, for the first time, that AGL confers significant protection against CLP-induced ALI, largely through PPARγ activation. The use of GW9662, a selective PPARγ antagonist, provided critical mechanistic insights, revealing that inhibition of PPARγ effectively reverses the beneficial effects of AGL on inflammatory cell infiltration, alveolar-capillary barrier integrity, and histopathological damage [58].

The reversal of anti-inflammatory effects upon co-treatment with GW9662 supports the hypothesis that AGL suppresses immune cell recruitment *via* PPARγ activation. Previous studies have established PPARγ as a negative regulator of inflammatory gene expression by antagonizing NF-κB and PTEN/β-catenin signaling pathways, which are often activated in sepsis-induced lung injury [62, 63]. Thus, our findings are consistent with the notion that AGL mitigates inflammation through PPARγ-dependent mechanisms.

Moreover, the abrogation of reduced lung inflammation, BAL fluid protein, and PPARγ expression by GW9662 underscores the role of PPARγ in maintaining endothelial and epithelial integrity during an inflammatory insult. This is supported by earlier reports showing that PPARγ activation enhances tight junction protein expression and reduces vascular permeability in models of lung injury [64]. One limitation of our study is the inability to perform mechanistic investigations using specific cell types, such as alveolar type 2 epithelial cells. To contextualize our findings at the cellular level, we refer to recent single-cell studies on traditional medicines in sepsis [65], which present analytic strategies that could be adapted to explore AGL’s PPARγ-dependent effects across distinct lung cell populations. Such approaches may help elucidate the cellular targets and mechanisms underlying AGL-mediated protection.

## 5 STUDY LIMITATIONS

Despite the promising findings, this study has certain limitations, including the lack of quantification of PPARγ protein and its downstream effects. Further downstream effectors of PPARγ signaling, such as PTEN/β-catenin or tight junction proteins (claudin-5, occludin, ZO-1), were not evaluated, which would have provided a more comprehensive mechanistic understanding. Future studies should include PPARγ functional studies, NF-κB p65 nuclear translocation, IκBα degradation, or expression of PPARγ-regulated antioxidant and anti-inflammatory genes.

## CONCLUSION

AGL confers protection against sepsis-induced ALI by modulating oxidative stress and inflammatory responses *via* PPARγ activation. These findings highlight the protective effects of AGL in sepsis-related pulmonary complications. Future studies should explore downstream pathways, synergy with other mechanisms, and AGL’s safety profile.

## Data Availability

All data generated or analysed during this study are included in this published article.

## LIST OF ABBREVIATIONS

AGL: Andrographolide
ALI: Acute Lung Injury
ARDS: Acute Respiratory Distress Syndrome
BAL Fluid: Bronchoalveolar Lavage Fluid
cDNA: Complementary DNA
CLP: Cecal Ligation and Puncture
GW: GW9662
HO1: Heme Oxygenase-1
H & E: Haematoxylin & Eosin
H_2_O_2_: Hydrogen Peroxide
IL: Interleukin
IP: Intraperitoneal
LPS: Lipopolysaccharide
MAPK: Mitogen-Activated Protein Kinase
MDA: Malondialdehyde
NLRP3: Nucleotide-Binding Domain, Leucine- Rich-Containing Family, Pyrin Domain- Containing-3
mTOR: Mammalian Target Of Rapamycin
NF-κB: Nuclear Factor Kappa Light Chain Enhancer Of Activated B Cells
NO: Nitric Oxide
Nrf2: Nuclear Factor Erythroid 2–Related Factor 2
O_2_^−^: Superoxide Ion Radical
PI3K: Phosphoinositide 3-Kinase
PBS: Phosphate Buffered Saline
PPAR: Peroxisome Proliferator-Activated Receptor
RAGE: Receptor for Advanced Glycation End Products
ROS: Reactive Oxygen Species
RT-PCR: Reverse Transcription Polymerase Chain Reaction
SE: Standard Error
SOD: Superoxide Dismutase
